# Cutaneous Anguillulosis During Immunotherapy for Metastatic Renal Cell Carcinoma

**DOI:** 10.3390/medicina61020339

**Published:** 2025-02-14

**Authors:** Pierre Cornillon, Marie Beguinot, Denis Maillet, Wafa Bouleftour, Yanis Bouqallaba, Pierre Flori, Pauline Corbaux, Guorong Li

**Affiliations:** 1 Department of Medical Oncology, North Hospital, CHU Saint Etienne, 42000 Saint Etienne, France; pierre.cornillon@chu-st-etienne.fr (P.C.); wafa.esquis@chu-st-etienne.fr (W.B.); yanis.bouqallaba@chu-st-etienne.fr (Y.B.); pauline.corbaux@chu-st-etienne.fr (P.C.); 2 Department of Medical Oncology, Medipole Lyon Villeurbanne Mutualist Clinic, 69100 Lyon, France; marie.beguinot@gmail.com; 3 Department of Medical Oncology, IMMUCARE, Centre Hospitalier Lyon Sud, Institut de Cancérologie des Hospices de Lyon, 69152 Pierre-Bénite, France; denis.maillet@chu-lyon.fr; 4 Laboratory of Parasitology, North Hospital, CHU Saint Etienne, 42000 Saint Etienne, France; pierre.flori@chu-st-etienne.fr; 5 Department of Urology, North Hospital, CHU Saint Etienne, 42000 Saint Etienne, France

**Keywords:** metastatic renal cell carcinoma, immunotherapy, skin toxicity, cutaneous anguillulosis, IRIS

## Abstract

Immunotherapy has been widely applied to treat metastatic renal cell cancer patients. Managing the side effects of immunotherapy can be a challenge. Here, we describe a rare presentation of cutaneous anguillulosis during immunotherapy. The patient experienced a rash eruption after receiving immunotherapy for metastatic renal cell cancer. The diagnosis of skin toxicity caused by immunotherapy was rejected after the failure of treatment by cortisone. The travelling history and a laboratory test confirmed the presence of anguillulosis infection. A diagnosis of cutaneous anguillulosis was established. Satisfactory treatment with ivermectin was achieved for cutaneous lesions. Immunotherapy was restored without any skin symptoms. We suggested the possible mechanism of cutaneous demonstration of immune inflammatory syndrome during the immunotherapy of a cancer patient with parasitic infection. It was concluded that a comprehensive search for previous infectious pathogens should be performed to ensure a correct diagnosis and timely treatment.

## 1. Introduction

The incidence of renal cell carcinoma has been increasing over the past few decades. Clear cell renal cell carcinoma (ccRCC) is a major histological subtype, accounting for about 80% of renal cell carcinomas. RCC is characterized by loss of the VHL gene, which results in increased angiogenesis. This mechanism has informed many advancements in targeted therapy in recent decades, such as tyrosine kinase inhibitors (TKIs), targeting the vascular endothelial growth factor receptor (VEGFR) and immune checkpoint inhibitors for advanced or metastatic renal cancers [1]. These medical treatments provide new hope for metastatic patients. Medicament treatment has become the main approach to treating patients with advanced, metastatic or recurrent RCC. However, many side effects have also been observed during treatment. The side effects can be challenging in routine practice. Therefore, the aim of this work was to present a rare case of cutaneous anguillulosis during immunotherapy for a patient with metastatic renal cell carcinoma. We suggested the possibility of cutaneous demonstration of immune inflammatory syndrome during immunotherapy for parasite-infected patients.

## 2. Case Report

**Figure 1:**
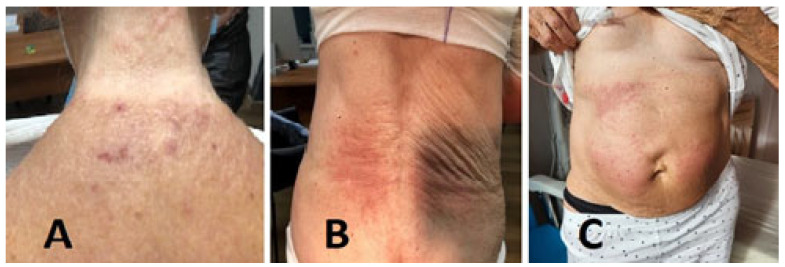
Patient’s skin presentation. Patient’s urticarial rash, presented on the neck (**A**), on the back (**B**), and on the abdomen (**C**).

This case report focuses on a 77-year-old female patient who was suffering from a papillary carcinoma of the right kidney in 2019. She was a retired teacher living in a small-sized city with a hobby of tourism. A partial nephrectomy was performed. Pathological diagnosis identified a papillary carcinoma with a TMN stage of pT1bR0 and a nuclear grade of 3. During a regular follow-up session, CT examination found a nodule in the pancreas in July of 2022. A tissue biopsy was performed. A routine pathological examination as well as immunohistochemical studies confirmed a pancreatic metastasis from the papillary renal cell carcinoma. The patient was treated with pembrolizumab plus axinitinib on 29 September 2022. After the first course of pembro/axitinib, the patient reported retroauricular itching with progressive extension to the back, arms, and abdomen. Ten days later, a rash was found to have erupted on the upper back, with progressive extension to the abdomen, arms and buttocks. Clinical examination revealed a fixed, widespread urticarial rash on the abdomen with respect to the umbilicus; the rash was well demarcated on the inner side of the arm, lower back and buttocks, upper back, and neck going up to the occiput. The skin eruption is depicted in Figure 1. The basic lesion was a fixed pruritic urticarial erythematous papule. No prior bubbles or mucosal damage were found.

The patient had an extensive travel history, having previously visited the Dominican Republic, Morocco (several times), Turkey, Tunisia, India, Thailand, Vietnam, Canada, Alsaka, and Kenya.

The possible skin toxicity of the immunotherapy was first considered, and the patient was treated with cortisone and antihistamine drugs. However, the skin symptoms were aggravated after cortisone treatment. The immunotherapy was stopped. A consultation by a dermatologist was requested. During the dermatological consultation, significant undulating blood and skin hyper eosinophilia of up to 4 G/L was found. A comprehensive search for pathogen infection was performed via blood or stool tests (Table 1). Assessment of hyper eosinophilia during the patient’s hospitalization in the dermatology department deemed the patient to be sero-positive with anguillulosis. A diagnosis of cutaneous anguillulosis was established by a dermatologist. The patient was treated with 200 µg/Kg ivermectin (two doses administered 10 days apart, given on an empty stomach for 2 days, to be repeated on day 10). After two and half months’ treatment, satisfactory results were obtained, and the patient described only very slight itching of the back. On clinical examination, the skin was well hydrated and there were no scratching lesions. An examination of the entire integument did not reveal any suspicious lesions. Thus, the diagnosis of eruption of parasitic origin was confirmed. Immunotherapy was restored without any skin toxicity.

## 3. Discussion

Anguillulosis or strongyloidiasis is a human parasitosis caused by a tiny strongyloides stercoralis [2,3]. More than 50 million people are infected with this disease worldwide. It is asymptomatic in 20–50% of cases [2,3]. Its clinical presentation is polymorphic, mainly intestinal, but sometimes extra-digestive. The parthenogenetic female parasites fix deeply in the duodenal mucosa and cause the disease. Its original infection cycle makes it possible for its presence in humans to perpetuate for decades, resulting in an inveterate disease responsible for persistent or sporadic eosinophilia. The durability of the disease is explained by the auto-reinfection cycle, in the absence of new contamination. The parasitological diagnosis requires the implementation of specific non-routine examinations intended only for the detection of living larvae, such as the classic Baermann extractive method [2,3]. Parasitological examinations of stools are of limited sensitivity due to the non-continuous elimination of eggs, larvae, or cysts. Treating anguillulosis has become easy and effective since the use of ivermectin, a molecule initially used in veterinary medicine, was introduced [4]. Serology and eosinophil count can be useful markers for treatment success [5].

Immunotherapy for metastatic RCC has become increasingly widespread. Diagnosing side effects may be challenging. Skin toxicity is common; this leads to eruption of a rash during immunotherapy in renal cancer patients, and treatment with cortisone and antihistamine is routine and effective [6,7]. However, in the present case study, this treatment aggravated skin symptoms. Other causes for the skin symptoms should be considered.

The diagnosis of anguillulosis is based on the identification of *S. stercoralis* larvae in the stool or the presence of antibodies, which is determined via serology [8,9]. The detection of larvae in the stools confirms the diagnosis, but the sensitivity is not optimal even with the methods of concentration. Serology is very useful for diagnosing *S. stercoralis* infection. The sensitivity of commercial ELISA tests is 80 to 90% and the specificity is 97%. Antibodies are detectable after two to four weeks of infection.

In our patient, significant undulating blood hyper eosinophilia was found when skin symptoms appeared. This observation led dermatologists to search for parasitic infection. The patient’s history of travelling to endemic areas and a laboratory test for parasitic infection clarified the diagnosis. The patient was serum-positive for anquillulosis. Treatment with ivermectin was successful in this patient.

**Figure 2:**
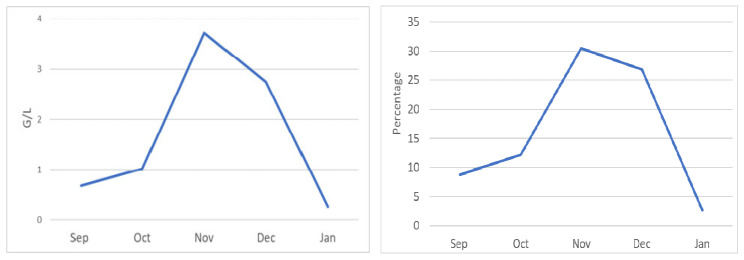
Hyper eosinophilia observed after immunotherapy. (**Left**) part indicates the count of eosinophils in G/L. (**Right**) part indicates the percentage of eosinophils.

This is an interesting and rare case. We thought that the possible mechanism of cutaneous presentation in this patient was related to immune reconstitution inflammatory syndrome (IRIS) [10]. IRIS was originally found in patients with human immunodeficiency virus (HIV) or hepatitis after starting antiviral treatment. IRIS is a heterogeneous inflammatory disorder and occurs as a state of an excessive and deregulated immune response to various infectious and non-infectious pathogens consecutive to the modification of immune status [11]. HIV-associated immune reconstitution disease (IRD) is the clinical presentation or deterioration of opportunistic infections that results from enhancement of pathogen-specific immune responses among patients responding to antiretroviral treatment [10,11,12]. The spectrum of infections now recognized as associated with IRD is expanding and includes a number of parasitic infections [8]. Reports of IRD related to parasitic infections are increasing in patients receiving antivirus treatment [12,13,14,15]. However, we have not found any reports of IRD-associated parasitic infection in cancer patients during immunotherapy. We thought that IRD associated with parasitic infection could occur in immunocompromised patients with cancer. When our patient, who was infected with a parasitic infection, anguillulosis, was treated with immunotherapy, IRD occurred with cutaneous presentation. Our suggestion was based on two main pieces of evidence: the timing of the appearance of skin symptoms and the timing of the appearance of hyper eosinophilia. During immunotherapy with pembrolizumab plus axinitinib, immune reaction is not instant, so immune-related adverse events do not appear immediately. It generally takes a minimum of several days or weeks for such responses to develop. The timing of the skin rash in the patient corresponded perfectly with the timing of the immune-related reaction. The appearance of symptoms was observed on day 10, with exacerbations between weeks 4 and 6, which was compatible with an immune boost. No hyper eosinophilia was observed at the beginning of the treatment, but the amount of eosinophils increased to 1.02 G/L at 2 weeks, then up to 3.72 G/L about 8 weeks after the administration (Figure 2). An obvious increase in the percentage of eosinophils was also demonstrated after the administration of immunotherapy (Figure 2). The timing of hyper eosinophilia matched perfectly with the symptoms. No episodes of parasitosis had occurred since the patient’s trips to Africa. No other moment of exposure was reported by the patient, other than this trip 10 years ago. Therefore, dormant parasitosis existed with reactivation at the time of immunotherapy. We emphasized the comprehensive search for infectious pathogens with blood and stool tests. In our case, only positive anguillosis was found, and other possible factors such as chronic virus infections were excluded. Early diagnosis of IRIS-associated diseases and appropriate treatment are fundamental to the patient’s prognosis. Close surveillance during immunotherapy initiation is necessary for finding IRIS. In the case of IRIS development, a detailed investigation of rare associated diseases including parasitic infection is of great importance for the treatment and the prognosis of these patients.

## 4. Conclusions

We reported the first case of cutaneous anguillulosis during immunotherapy for metastatic renal cell carcinoma. A comprehensive search for previous infectious pathogens should be performed to ensure a correct diagnosis and timely treatment.

## Figures and Tables

**Table 1:** Comprehensive search for pathogens.

Type	Examination Name	Result
Blood—Virology	HIV1, HIV2, Hepatitis B, Hepatitis C	Negative
Blood—Antibody	Anti-Skin, Anti-HuD, Anti-Yo, Anti-Ri, Anti-CV2, Anti-Amphiphysin, Anti-Ma1, Anti-Ma2, Anti-GAD65, Anti-Sox1, Anti-Tr, Anti-Zic4, Anti-Titin (MGT30), Anti-PKCgamma, Anti-Recoverin	Negative
Blood—Parasitology Stool—Parasitology	Anguillulosis, Bilharziasis, Distomatosis, Filariosis, Toxocarosis, Trichinosis Check for eggs, cysts, or larvae of adult parasites	Anguillulosis + Negative

## Data Availability

The data presented in this study are available in the article.
